# Domestication and ontogeny effects on the stress response in young chickens (*Gallus gallus*)

**DOI:** 10.1038/srep35818

**Published:** 2016-10-26

**Authors:** Maria Ericsson, Per Jensen

**Affiliations:** 1 AVIAN Behavioural Physiology and Genomics Group, IFM Biology, Linköping University, Linköping, Sweden

## Abstract

Domestication is thought to increase stress tolerance. The connection between stressor exposure, glucocorticoids and behavioural responses has been studied in adults, where domestication effects are evident. Early stress exposure may induce detrimental effects both in short-and long term. Previous research has reported a lack of glucocorticoid response in newly hatched chickens (*Gallus gallus*), whereas others have found opposite results. Hence it remains unclear whether the HPA-axis is functional from hatch, and if domestication has affected the early post-hatch ontogeny of the stress response. Our aims were to investigate the early ontogeny of the HPA-axis and characterize behavioural and hormonal stress responses in ancestral Red Junglefowl and in two domestic layer strains. Plasma corticosteone and behavioural responses before and after physical restraint was measured on day one, nine, 16 and 23 post hatch. The results showed significant increases of corticosterone after stress in all three breeds at all the different ages. The HPA-response decreased with age and was lower in Red Junglefowl. Behavioural responses also decreased with age, and tended to be stronger in Red Junglefowl. In summary, the HPA-axis is reactive from day one, and domestication may have affected its development and reactivity, alongside with related behaviour responses.

During domestication animals have adapted to environments and circumstances provided by humans^1^, resulting in considerable changes in morphology, behavior and physiology. A main driving force in the domestication process has been suggested to be increased tolerance to stress. Comparisons between domesticated animals and their ancestors have demonstrated increased stress tolerance in domesticates in a range of species^2^,^3^,^4^,^5^. This includes chickens, where the wild ancestor, the Red Junglefowl (RJF), shows more fear related behavior^6^ and a different pattern of stress recovery, with larger immediate behavioural change after an acute stressor exposure and a faster return to baseline compared to a domestic breed^7^. Furthermore, domestication effects have been found in chickens both with respect to the corticosterone response to restraint as well as to the recovery patterns, where the RJF showed a more pronounced increase with a faster return to baseline^7^. Similar stress response patterns between wild and domestic counterparts have been observed in other species, for example in rainbow trout and Eurasian perch^8^ and in foxes^9^.

Stress is closely linked to increased activity in the hypothalamic-pituitary-adrenal (HPA)-axis, and thereby to increased levels of glucocorticoids including cortisol and corticosterone (CORT)^10^. Upon activation, energy resources are mobilized and sets the animal in an “emergency life-history stage”^11^, preparing the animal for taking action towards a potential treat. Whereas glucocorticoids are essential for normal development, variable levels during brain maturation may induce behavioural and physiological deficiencies^12^. During a short post-natal period, a stress-hyporesponsive period (SHRP) been reported for some species, where the HPA-axis is immature and environmental stressors do not cause release of cortisol/corticosterone. The presence of an SHRP is evident in several rodent species^13^,^14^,^15^, and possibly also in dogs^16^. According to the developmental hypothesis in birds^17^, the SHRP should be more pronounced in altricial species but less clear in precocial hatchlings (Blas in ref. 18). Hence, the highly precocial chicken (*Gallus gallus*) would be expected to show a weak SHRP.

However, previous data on HPA-axis activity in newly hatched chickens are conflicting. Circulating baseline levels of glucocorticoids are detectable during embryonic development^19^, indicating a pre-hatch maturation of the adrenal glands. Furthermore, partial or constant modification of incubation temperature modifies the pattern of HPA-axis reactivity post hatch^20^. Injection of ACTH on day one post hatch significantly elevated the levels of circulating CORT^21^,^22^, whereas bone fracturing failed to evoke adrenal steroid responses^21^. Elevated post stress CORT levels were, however, evident both during the embryonic stage and on day seven post hatch. A later replicate of the experiment, however, showed unresponsiveness to ACTH injection on day one^21^. Another experiment showed that cold stress did not trigger an HPA-axis response on day one^23^, supporting the presence of a stress-hyporesponsive period in chickens. Brief restraint did however trigger the HPA-axis^24^. Hence, previous results are conflicting and it remains unclear how the HPA-axis develops post hatch in chickens, as well as whether this has been modified by domestication.

In poultry production, day-old chicks are exposed to a range of potential stressors (rough human handling, sex-sorting, transportation in trucks etc.). Whilst the existence of an SRHP would indicate that chicks are perhaps unresponsive to the management procedures on day one, the conflicting data presented above opens the possibility that the process may indeed be stressful and could result in short- and long term negative effects on behavior and physiology.

The aim of this project was to obtain a cohesive view of the early post-hatch ontogeny of the HPA-axis reactivity to stress in chickens, including behavioural and hormonal stress reactions, and the way in which this may have been modified by domestication. Our hypothesis was that chickens would show a well-developed HPA-axis response to physical restraint already on day one post-hatch, and that domesticated chickens would have a less acute response at all test ages. To account for strain differences, we included two different domesticated White Leghorn layer strains, one relatively un-selected, and one commercial hybrid, heavily selected for high egg production.

## Results

To assess hormonal responses to acute stress, chicks from the two White Leghorn strains and from ancestral Red Junglefowl were stressed by being physically restrained in a mesh cloth bag for 10 min at different ages from hatch to 23 days of age. Blood plasma corticosterone was measured in a baseline blood sample and after restraint. We also recorded the behaviour of chicks in an arena where they were moving freely and had access to social contact both before and after the restraint period. A univariate GLM showed no significant sex differences (Range of parameters: F = 3.87 − 0.001; df = 1, 5 − 1, 8; all P > 0.05) in any behaviour variables, or in CORT levels either on baseline levels or after restraint at any of the sample ages, and sex was therefore not considered further in the analyses.

### Corticosterone

**Figure 1:**
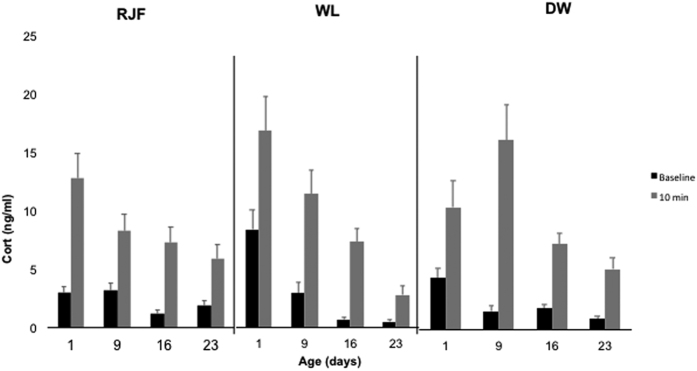
Means for corticosterone levels. The bars show average CORT levels before and after physical restraint in the three breeds (RJF = Red Junglefowl, SLU13 = White Leghorn research strain, DW = Dekalb White) at all sample ages. Error bars show standard errors.

All birds responded to the physical restraint with a significant increase of CORT after 10 minutes (10 MIN), regardless of breed (t = −5.35 − 3.03; df = 7 − 9, all P > 0.05. This increase was seen at all sampling ages in all strains (Fig. 1) except for SLU13 on day 1 where a tendency was seen (t (9) = −2.06; P = 0.069.

There was a significant difference in baseline levels of CORT between the sample ages, which became lower with increasing age (Wald χ^2^ = 67.34, df = 3, P < 0.001), and a tendency for effect of breed (Wald χ^2^ = 4.95, df = 2, P = 0.084). There was a significant interaction between breed and age on baseline CORT concentrations, reflecting that baselines were higher in SLU13 and DW at day one (Wald χ^2^ = 35.33, df = 6, P < 0.001).

Also the post-restraint levels of CORT were significantly different between sample ages, again generally decreasing with increasing age (Wald χ^2^ = 46.02, df = 3, P < 0.001), but there was no overall breed effect (Wald χ^2^ = 0.71, df = 2, P = 0.70). A significant breed * age interaction was observed also on the post-restraint levels, again reflecting higher levels in the domesticated breeds at younger ages (Wald χ^2^ = 15.89, df = 6, P = 0.041).

The relative increases of CORT levels (post-stress–baseline) were calculated and there was a significant effect of age (Wald χ^2^ = 15.91, df = 3, P = 0.001), where responses were stronger in younger chicks. There was no main effect of breed (Wald χ^2^ = 0.68, df = 2, P = 0.71). However, again a significant interaction between breed and age was revealed (Wald χ^2^ = 12.57, df = 6, P = 0.050), reflecting a larger increase in SLU13 at earlier ages.

To assess behavioral responses to acute stress, activity and social coherence were assessed before and after a brief event of physical restraint. Baseline behavior was recorded for 30 minutes in an arena where the chick could move freely and had access to social contact with group-mates through a wire-mesh in one end of the arena. Thereafter, the bird was restrained in a mesh cloth bag for 3 minutes. Immediately following this, it was let back into the test arena and the behavior recordings continued for 30 minutes, enabling a comparison of pre-and-post stress behaviour. The behaviours measured were the total distance moved in the arena and the duration spent in the zone next to the companion birds.

### Behaviour

**Figure 2:**
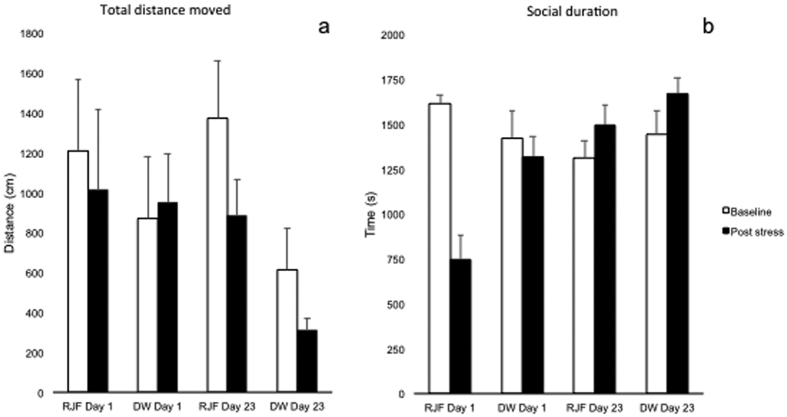
Mean values of behavioural data. (**a**) Mean values (+/− SEM) for “Total distance moved” before and after restraint stress for each of the breeds at one and 23 days of age. (**b**) average time (+/− SEM) spent in the social zone next to the companion birds at one and 23 days of age. RJF = Red Junglefowl, DW = Dekalb White.

The total distance moved in the arena on day one revealed no significant main effects of breed (F_1,68_ = 0.08; P = 0.78) or stress (pre- or post-stress) (F_1,68_ = 0.20; P = 0.67) (Fig. 2a). However, a significant breed * stress interaction was seen (F_1,68_ = 6.06; P = 0.016), where the RJF moved more before and less after restraint, while the opposite was seen in the DW.

On day 23, a significant breed effects was seen (F_1,64_ = 13.22; P = 0.001), showing a higher activity in the RJF both before and after the restraint stress. A significant overall stress effect (F_1,64_ = 4.77; P = 0.033) was also seen, but there was no significant time * stress interaction (F_1,64_ = 0.22; P = 0.64). When investigating the baseline change over time, comparing day one and 23, a tendency towards a breed effects was seen (F_1,64_ = 3.49; P = 0.066) where the RJF was had a higher activity level at both ages compared to DW. No age effect (F_1,64_ = 0.21; P = 0.65) or age * breed (F_1,64_ = 0.39; P = 0.53) effect were observed.

With respect to the behavioural change between pre and post stress (post stress minus baseline), neither results from day one nor 23 showed significant breed effects when tested separately at each age (Day one Wald χ^2^ = 0.27, df = 1; P = 0.61); day 23 (Wald χ^2^ = 0.67, df = 1; P = 0.41)). Comparing the same data between day one and 23, there was no significant age effect (F_1,64_ = 1.88; P = 0.18), breed effect (F_1,64_ = 0.82; P = 0.37) or age * breed interactions (F_1,64_ = 0.027; P = 0.87).

For “time spent in social zone”, on day one, no significant effects were observed of stress (F_1,68_ = 0.43; P = 0.51) or breed (F_1,68_ = 0.51; P = 0.48), but there was a significant stress * breed interaction (F_1,68_ = 10.06; P = 0.002) (Fig. 2b). Both breeds spent on average less time in the social zone after stress exposure, but the decrease in time was more clear in RJF. For the same behavior on day 23, no significant stress effect was observed (F_1,64_ = 0.06; P = 0.81), but there was a significant breed effect (F_1,64_ = 4.70; P = 0.034) where RJF spent less time in the social zone compared to the DW. On day 23, there was a tendency for an interaction between breed and stress (F_1,64_ = 3.04; P = 0.086), indicating a tendency for DW to spend more time in the social zone post stress. With respect to the baseline behavioural change over time, comparing day one and 23, no significant age effect (F_1,64_ = 1.17; P = 0.28), breed effect (F_1,64_ = 0.02; P = 0.90), or age * breed interactions (F_1,64_ = 1.58; P = 0.21) were seen.

With respect to the change in duration in social zone between pre and post stress, there was a significant breed effect on day one Wald χ^2^ = 11.3, df = 1; P = 0.001) where RJF had a clear decrease in behavioural change compared to DW. On day 23, no difference was observed in this respect Wald χ^2^ = 0.06, df = 1; P = 0.8 When analyzing the variable post stress minus pre stress between day 1 and 23 on duration in the social zone, significant effects were observed of age (F_1,64_ = 24.56; P < 0.000), breed (F_1,64_ = 7,98; P = 0.006), and of the interaction between the both (F_1,64_ = 6.48; P = 0.013), where the difference was larger in RJF compared to the DW, an effect almost entirely attributed to the difference observed on day one.

## Discussion

Our results show that the HPA-axis was responding to physical restraint stress already on day one post hatch in chickens, but there was still a significant development of the response at least until day 23. Furthermore, baseline levels as well as post-restraint levels of corticosterone differed between domesticated chickens and ancestral Red Junglefowl, implying that domestication has modified the development and reaction of the HPA-axis. Both pre- and post-stress CORT levels decreased with age, indicating an ongoing maturation of the system. Behavioural changes as a result of the stress were observed in both breeds, and also in this respect, there was a development over time with indications of domestication effects.

Contradicting results has previously been reported for the reactivity of the HPA-axis immediately after hatch in chickens^21^,^22^,^23^,^24^. Hence, the present results contribute to a more coherent picture, using modern analysis methods, alongside with detailed behavioural data. Some of the potential problems in earlier studies, which may explain previous ambiguous results with respect to CORT responses on day one, include that baseline levels and the post-stress levels have sometimes been determined in different groups, precluding accounting for individual variation in baselines^23^,^24^, or that studies have used pooled samples^25^ which could also lead to individual variation masking the actual effects. In the present study, pre- and post stress levels were measured from the same individual (however different individuals were used in the HPA-axis reactivity and behaviour test at the different ages). Noticeable is the very large increase of CORT elicited by ACTH injection on day one found by Decuypere *et al*.^22^. The authors found strain-dependent CORT-levels 30 minutes after ACTH injection to be between 75 and 110 ng/ml, while the CORT levels in the present data did not exceed 25 ng/ml. This could be due to different analysis methods, but more likely is that the injected ACTH dose was higher than the levels released from the pituitary upon environmental stress. We can therefore conclude that a hypo-responsive stress period does not exist in chickens, and we can safely assume that the birds will be responsive to stressful experiences immediately after hatch. Our results further corroborate the developmental hypothesis^17^, claiming that precocial and altricial bird species differ in their ability to respond to environmental perturbations depending on how well-developed they are at hatch.

Exposure to stress at an early age can profoundly influence different aspects of development. This has been thoroughly investigated in rodents and can give rise to detrimental effects later in life, such as increased visceral sensation and altered bowel microbiota^26^, development of a hyper-responsive HPA-axis and negative behavioural effects (as reviewed by for example Lupien *et al*.^12^ or Levine *et al*.^27^). Similar effects have been found in chickens, where both immediate, long-term, and transgenerational effects have been observed in a range of behavioural and physiological traits^28^,^29^,^30^.

Previous research has shown that already from embryonic day ten, circulating levels of glucocorticiods can be detected in chickens^19^ and baseline plasma glucocorticoids show two significant peaks in the embryo, the first around embryonic day 14–16 where equal amounts of cortisol and corticosterone can be detected, and the other at day 20 just before hatch, when predominantly corticosterone is secreted, indicating a maturation of the system during the embryonic stage (for a review, see ref. 31). This supports our present results, and indicates that most of the maturation of the HPA-axis necessary for a distinct stress response occurs before hatch. However, our results showed a significant pattern of decreased baseline and stress-induced CORT levels over the observed sampling ages, indicating an ongoing maturation of the neuroendocrine system post-hatch. It should be noted that the power to detect sex differences was small in the experiment, due to the relatively few animals of each sex in each breed and age group.

Previous studies have shown significant strain differences in HPA-reactivity^32^. Our results show similar CORT levels in the two different domestic strains, but a more pronounced alteration in the wild ancestor, especially at day 23 when the HPA-axis has seemingly stabilized and the CORT response is similar to the levels of adult WL and RJF^7^. This suggests that the differences in stress response between strains is not only seen in adult individuals, but is evident already from hatch. Contrary to our hypothesis, we found that domesticated birds had a stronger HPA-reactivity on day one than the wild ancestors. One could expect that domesticated chickens have been selected for increased stress tolerance, in particular during the early ages, since this is associated with considerable stress under commercial conditions. As mentioned above however, an indication of an ongoing maturation of the HPA-axis was seen and perhaps the typical domestication patterns does not apply until full maturation of the system. A full understanding of the stress response would require investigating also receptor activity and feed-back systems in different parts of the brain and the endocrine system. It remains possible that the system is less responsive in domesticates in spite of the higher CORT response, perhaps due to variations in receptor densities. This would seem to be supported by the fact that the behavioral responses were stronger in the RJF in spite of lower CORT response (discussed further below). For practical reasons, blood was drawn from different veins depending on age. Blood is normally taken from the brachial vein but it is not visible in 1-day old chicks, and blood sampling from the jugular vein in older birds is inconvenient both for experimenter and for the bird. Drawing blood from different veins could potentially confound the day-one data.

The behavioural data are in line with previous findings on domestication effects in chickens, where RJF are generally more explorative and active compared to domestic breeds^6^. On day one after hatch, RJF associated less with conspecifics and were less active following stress than the domesticated DW (Fig. 2a,b). However, this difference had decreased or disappeared on day 23. In general, the behavior on day 23 reflects previously reported adult behavioural reactions in chickens, where acute stress usually leads to an increase in sociality^33^. The larger similarities to adult behavior patterns seen on day 23, compared to day one, indicate that stress related behaviors undergo maturation and become more stable with age, similar to the HPA-axis activity. Furthermore, the breed differences indicate that selection for production traits during domestication has altered the behavioral stress coping systems. The DW birds were incubated until day 18 at a different location compared to the RJF and the SLU13, which may have affected the findings. The DW however follow similar developmental patterns post hatch as the two other breeds which indicates limited effects of incubation differences.

Overall, the present results clearly indicate that the HPA-axis is responsive to acute stress in day old chicks. This may also be directly related to the behavioral effects observed, since coping styles and personalities are affected by circulating CORT levels. For example, a more bold personality was seen in Florida Scrub-jays exposed to elevated glucocorticoid levels on day 11 post hatch^34^ and steroids also affect memory and learning abilities^35^,^36^.

Under commercial production conditions, domestic chickens are hatched in highly artificial environments and are exposed to numerous potential stressors during their first days of life, which may be exacerbated by the absence of a mother^37^. Early stress in chickens may induce larger lateralization in the hippocampus^38^ and can contribute to the development of abnormal behaviours such as feather pecking and cannibalism^39^,^40^,^41^ Our results therefore have strong implications for welfare considerations relating to commercial hatchery procedures.

In conclusion, this study shows that there is a functioning but developing HPA-axis in one-day-old chicks. Furthermore, differences in glucocorticoid response patterns and in stress induced behaviour between the breeds suggest that domestication has altered the stress response. On day 23, behaviour as well as CORT reactions were more similar to the previously observed adult patterns. Further on-farm studies are required to properly evaluate possible long-time effects of stressful hatchery management procedures. Such research would be of high relevance for understanding welfare in commercially bred laying hens.

## Materials and Methods

### Ethical statement

All experimental protocols were carried out in accordance with the relevant guidelines and regulations. The experimental protocols were approved by the Linköping Council for Ethical Licensing of Animal Experiments, ethical permit no 122-10.

### Animals and rearing conditions

Birds from three different breeds of chickens were used: ancestral Red Junglefowl (RJF), the White Leghorn research strain SLU13 originally stemming from an outbred populations and then bred for research purposes and egg mass, and the commercial layer hybrid Dekalb White (DW). For details on the backgrounds of the SLU13 and the RJF, see ref. 42. The eggs from the RJF and SLU13 were incubated from day one of incubation in our research facilities in a Marsalles 25 DIGIT incubator. The incubation temperature was set to 37.8 °C and 55% relative humidity. On day 18 of incubation, the settings were changed to hatch settings: 37.5 °C and 65% relative humidity. The DW were obtained as fertilized eggs from a commercial supplier (Swedfarm, Linghem, Sweden) and transported for approximately 20 minutes to our research facility in a portable incubator maintaining 37 °C on day 18 of incubation. They were then hatched in the same incubator as birds from the other two strains under identical circumstances as mentioned above.

All birds were raised in identical pens measuring 1 × 1 m, equipped with feed and water *ad libitum* and with wood shavings on the floor. The RJF were hatched in two separate batches, one for CORT measures and one for later behavior tests. For the HPA-axis reactivity test, the RJF and SLU13 where hatched two days apart to allow testing the birds within the same time span during the sample days. The DW was hatched and tested for CORT values at a separate occasion. The DW and the RJF hatched for behavioural testing were hatched two days apart in order to allow efficient use of test arenas.

### Behaviour recordings

For behaviour recordings, only the RJF and the DW strains were included. The behavior was recorded on post-hatch day one and 23, using the same birds at both ages. 18 birds from each breed were used, however due to mortality, only 16 RJF remained for testing on day 23. The behavior test was designed to record the behavior of individual chicks in a free-running situation with options for maintaining close social contact with peers. The focal individual was placed in an arena measuring 0.9 × 0.4 m, equipped with a beige rubber mat, feed and water. Cameras and LED-lights were attached to the roof. Two familiar birds were placed in a smaller compartment at the end part of the arena, separated by wire mesh from the test arena. Prior to the onset of behavior recordings, the birds were allowed to habituate for 30 minutes. Then the cameras were turned on and 30 minutes of automated video tracking took place to obtain baseline activity. The bird was then taken out of the arena and physically restrained for 3 minutes in the same way as explained below (*section HPA-axis reactivity*). Following that, the bird was placed back in the arena and an additional 30 minutes of post stress video tracking took place. The video analysis was carried out with EthoVision 10 (Noldus Information Technology bv, Waeginingen, The Netherlands). The arena was divided into 4 equally sized zones. The variables measured were total distance moved, and time spent in the social zones (the zone closest to the companion birds).

### HPA-axis reactivity

To quantify the HPA-axis reactivity, physical restraint was applied and CORT levels pre- and post-stress were assessed on day 1, 9, 16 and 23 post hatch. Different animals were tested at each age to avoid habituation to the restraint procedure, however within age group, the same bird were tested at both baseline and at 10 min. For RJF, 9 or 10 animals were tested at each sample age (n = 38), for SLU13, ten animals of each breed were tested at each sample age (n = 40 per breed). For DW, 8 or 9 animals were tested per sample age (n = 33). The number of animals divided by age, breed and sex in each part of the project is outlined in Table 1.

All test instances started at 13:30 h +/− 15 minutes, to minimize diurnal variations in CORT levels. The birds were collected in darkness from their home pens and within 3 minutes, a baseline blood sample of 150 μl was collected in heparin-coated tubes (Microvette, Sarstedt). In 1-day-olds, samples were obtained from the jugular vein, and from the older birds from the brachial (wing) vein. The birds were then manually restrained by being placed in a fine-meshed bag for 10 minutes, which has previously been shown to evoke a stress response (Ericsson, 2014), where after a post-stress blood sample (10 MIN) was collected in the same manner as the baseline sample. The samples were stored on ice and immediately centrifuged on 3000 rpm for 20 minutes. The plasma was then stored in −20 °C until further analysis. After CORT ELISA (see next paragraph), two samples failed to detect any CORT and was therefore excluded.

### Corticosterone ELISA

For the quantitative determination of CORT concentrations, a commercial corticosterone-specific ELISA-kit was used (ENZO Life Sciences). The samples were tested in duplicates and in accordance to the product protocol (online manual: http://static.enzolifesciences.com/fileadmin/files/manual/ADI-900-097_insert.pdf). The sensitivity of the assay was 26.99 pg/ml. Inter-assay and intra-assay coefficients of variation were 8.4% and 10.7% respectively.

### Statistics

The statistical calculations were carried out in IBM SPSS Statistics 23. A univariate General Linear Model was used to analyze sex differences. A paired samples t-test was used for comparing baseline CORT levels with the corresponding 10 MIN samples. To determine factors affecting HPA-axis reactivity, a Generalized Linear Model was used with breed and age (days) included in the model. The increase in CORT level after stress was calculated by subtracting post-stress from pre-stress values. The probability distribution was set to “Normal” and the link function was set to “Identity”. For all Generalized Linear Model analyses, the Omnibus test indicated an adequate model; (Baseline CORT levels: (Likelihood Ratio χ^2^ = 78.28, df = 11; P <0.0001), 10 MIN CORT levels: (Likelihood Ratio χ^2^ = 50.53, df = 11; P < 0.0001), increase of CORT from baseline to 10 min: (Likelihood Ratio χ^2^ = 25.49, df = 11; P =0.008)).

For the behavior analysis, the data followed a Poisson distribution. The data were transformed prior to statistical analysis, adding 1 to every individual behavioural value (resulting in values > 0). Two different Generalized Linear Mixed Models were performed, one with pre/post stress as within subject factor and breed as between-subject factor and individual as random effect, the second one with baseline levels on day one and day 23 as within subject factor, breed as between-subject factor and individual as random effect.

As a measure of behavioural change due to the stress treatment, post stress values minus baseline values were calculated and turned out normally distributed. The breed differences, as well as the difference between pre and post stress behavioural change over time (day one compared to day 23), were analyzed with Generalized Linear Models. The Omnibus test showed: Total distance moved day 1: (Likelihood Ratio χ^2^ = 0.67, df = 1; P = 0.41) day 23: (Likelihood Ratio χ^2^ = 0.27, df = 1; P = 0.61) and duration social zone day 1: (Likelihood Ratio χ^2^ = 9.78, df = 1; P = 0.002), day 23: (Likelihood Ratio χ^2^ = 0.064, df = 1; P = 0.80).

## Additional Information

**How to cite this article**: Ericsson, M. and Jensen, P. Domestication and ontogeny effects on the stress response in young chickens (*Gallus gallus*). *Sci. Rep.* **6**, 35818; doi: 10.1038/srep35818 (2016).

**Publisher’s note:** Springer Nature remains neutral with regard to jurisdictional claims in published maps and institutional affiliations.

## Figures and Tables

**Table 1:** Number of animals included in the cort analysis per breed and per age group.

Breed	Day 1 (n)	Day 9 (n)	Day 16 (n)	Day 23 (n)
**RJF**	9 (5/3/1)	9 (3/4/2)	10 (7/3/0)	10 (3/5/2)
**WL**	10 (7/2/1)	10 (5/5/0)	10 (3/7/0)	10 (6/4/0
**DW**	8 (5/2/1)	8 (5/3/0)	8 (6/1/1)	9 (6/3/0)

The first number show the total number of animals. In brackets are (Females/Males/Unknown sex).
